# Maternal Fluoxetine Exposure Alters Cortical Hemodynamic and Calcium Response of Offspring to Somatosensory Stimuli

**DOI:** 10.1523/ENEURO.0238-19.2019

**Published:** 2019-12-19

**Authors:** Rachel M. Rahn, Susan E. Maloney, Lindsey M. Brier, Joseph D. Dougherty, Joseph P. Culver

**Affiliations:** 1 Department of Radiology, Washington University School of Medicine, St. Louis, MO 63110; 2 Department of Genetics, Washington University School of Medicine, St. Louis, MO 63110; 3 Department of Psychiatry, Washington University School of Medicine, St. Louis, MO 63110; 4 Intellectual and Developmental Disabilities Research Center, Washington University School of Medicine, St. Louis, MO 63110; 5 Department of Biomedical Engineering, Washington University, St. Louis, MO 63110; 6 Department of Physics, Washington University, St. Louis, MO 63110

**Keywords:** calcium imaging, cerebral hemodynamics, fluoxetine, serotonin, somatosensory

## Abstract

Epidemiological studies have found an increased incidence of neurodevelopmental disorders in populations prenatally exposed to selective serotonin reuptake inhibitors (SSRIs). Optical imaging provides a minimally invasive way to determine if perinatal SSRI exposure has long-term effects on cortical function. Herein we probed the functional neuroimaging effects of perinatal SSRI exposure in a fluoxetine (FLX)-exposed mouse model. While resting-state homotopic contralateral functional connectivity was unperturbed, the evoked cortical response to forepaw stimulation was altered in FLX mice. The stimulated cortex showed decreased activity for FLX versus controls, by both hemodynamic responses [oxyhemoglobin (HbO_2_)] and neuronal calcium responses (*Thy1*-GCaMP6f fluorescence). Significant alterations in both cortical HbO_2_ and calcium response amplitude were seen in the cortex ipsilateral to the stimulated paw in FLX as compared to controls. The cortical regions of largest difference in activation between FLX and controls also were consistent between HbO_2_ and calcium contrasts at the end of stimulation. Taken together, these results suggest a global loss of response signal amplitude in FLX versus controls. These findings indicate that perinatal SSRI exposure has long-term consequences on somatosensory cortical responses.

## Abstract (precis)

Epidemiological studies have found an increased incidence of neurodevelopmental disorders in populations prenatally exposed to selective serotonin reuptake inhibitors (SSRIs). Optical imaging provides a minimally invasive way to determine if perinatal SSRI exposure has long-term effects on cortical function. Herein we probed the functional neuroimaging effects of perinatal SSRI exposure in a fluoxetine (FLX)-exposed mouse model.

## Significance Statement

Use of selective serotonin reuptake inhibitors (SSRIs) by pregnant women has increased in the past decades, raising questions regarding the long-term effects of SSRI usage during pregnancy on offspring. In order to isolate the effect of perinatal SSRI exposure from genetic variables or a maternal psychiatric diagnosis, we used *in vivo* functional neuroimaging to examine adult cortical function both at rest and in response to a somatosensory input in a mouse model of perinatal SSRI exposure. Our mouse model displayed no global disruption of brain function at rest when compared to controls, while its cortical response to a somatosensory stimulus was reduced, as measured by both hemodynamics and excitatory calcium signaling.

## Introduction

The serotonin (5HT) system and its proper development are critical to typical neurologic function, and implicated in a variety of neurodevelopmental processes and psychiatric conditions (Vitalis and Parnavelas, 2003; O’Connell et al., 2018). 5HT modulation is a common target for pharmaceuticals such as selective serotonin reuptake inhibitors (SSRIs), which are widely used for the treatment of depression and anxiety (Linde et al., 2015), including in an increasing proportion of pregnant women (Cooper et al., 2007; Mitchell et al., 2011).

It is known that 5HT also plays key roles in brain development (Vitalis and Parnavelas, 2003; Sodhi and Sanders-Bush, 2004). At fetal stages, 5HT receptors are already localized in the human thalamus (Wai et al., 2011), and are required for proper development of sensory gating circuits (McCormick and Bal, 1994). The 5HT transporter is also transiently expressed in thalamocortical axons during development (Lebrand et al., 1996; Bonnin et al., 2007), and altered 5HT activity, including that achieved with early SSRI exposure, has been linked to morphologic changes in the rat somatosensory cortex and its thalamocortical afferents (Lee, 2009). However, an important question remains whether the presence of SSRIs during brain development alters somatosensory connectivity specifically, or brain development generally, in a manner that has long-term consequences.

Despite the increase in SSRI usage in pregnant women, the full long-term effects of perinatal SSRI exposure are unclear. Long-term developmental and emotional alterations, such as increased anxiety (Lupattelli et al., 2018) and depression (Malm et al., 2016; Liu et al., 2017) diagnoses, have been observed in populations prenatally exposed to SSRIs. Altered behavioral responses to pain (Oberlander et al., 2002, 2005) and poor neonatal adaption (Olivier et al., 2013) have also been noted in SSRI-exposed infants. There is a lack of consensus regarding whether some behavioral alterations can be directly linked to prenatal SSRI exposure or if maternal psychiatric stress plays a major role (Oberlander et al., 2007; Hanley et al., 2015; Lupattelli et al., 2018). There also exists a lack of data available regarding postpubertal effects. Because human behavior is difficult to directly link to neural perturbations, more immediate quantifiable phenotypes might be obtainable through brain imaging.

Human brain imaging outcomes suggest both structural and functional abnormalities in the perinatally-exposed population. Compared to controls or infants with depressed mothers, neonates with SSRI-treated mothers displayed increased gray matter volume and white matter connectivity in the amygdala and insula (Lugo-Candelas et al., 2018). Functional neuroimaging studies observed connectivity increases in infants’ auditory resting-state network following prenatal SSRI exposure (Rotem-Kohavi et al., 2019) and reduced interhemispheric connectivity in EEG studies (Videman et al., 2017). However, functional neuroimaging studies have not yet explored the effects of perinatal SSRI exposure after the neonatal period or used task-based studies to determine potential alterations in sensory inputs. Human studies of perinatal SSRI exposure are also complicated by the genetic and environmental heterogeneity that is intrinsic in any human population, as well as the confounding influence of maternal diagnosis.

To ameliorate some of these problems, mouse models provide an avenue to study the biological impact of one isolated manipulation such as perinatal SSRI exposure in a controlled genetic background and environment. In rodent models, early developmental SSRI exposure has been associated with increased depression-like and anxiety-like behavior (Hansen et al., 1997; Ansorge, 2004; Gingrich et al., 2017), as well as alterations in somatosensory-related behaviors (Lee, 2009; Maloney et al., 2018). Early postnatal SSRI exposure in rats reduced dendritic complexity of Layer V pyramidal neurons in motor cortex (Lee and Lee, 2012) and of thalamocortical afferents and Layer IV spiny stellate neurons in somatosensory cortex (Lee, 2009). Prenatal fluoxetine (FLX) exposure also reduced dendritic complexity of layer 2/3 pyramidal neurons in P6–P9 mouse somatosensory cortex and remained even up to 16 months of age (Smit-Rigter et al., 2012). There however remains a lack of functional neuroimaging literature examining SSRI exposure models that bridges the gap between morphologic and behavioral abnormalities.

We therefore imaged a mouse model of perinatal SSRI exposure *in vivo* to characterize adult cortical function both at rest and following sensory input. Because of the close relationship between 5HT, SSRIs, and thalamocortical projection development, we examined whether functional neuroimaging of a perinatal SSRI exposure model would uncover functional disruption globally as well as specifically in somatosensory cortex, a region uniquely vulnerable to SSRIs. We found that our mouse model of early SSRI exposure displayed resting-state functional connectivity similar to controls, yet exhibited a reduced cortical response to somatosensory stimulation as quantified by both hemodynamics and excitatory calcium signaling.

## Materials and Methods

### Animal preparation

All procedures using mice were approved by the Washington University Institutional Animal Care and Use Committee and conducted in accordance with the approved Animal Studies Protocol. Mice were housed in translucent plastic cages measuring 28.5 × 17.5 × 12 cm with corncob bedding and standard lab diet and water freely available. The colony room lighting was a 12/12 h light/dark cycle, and the room temperature (∼20–22°C) and relative humidity (50%) were controlled automatically.

Following the previously published description of a perinatal SSRI exposure model (Maloney et al., 2018), mouse dams were exposed to the SSRI FLX through drinking water (48 mg/d in 1% saccharin water) beginning immediately before gestation and ending at pup postnatal day 14. This exposure time period and dosage were chosen to replicate the effects of usage of the maximum recommended human dose of FLX (Marken and Munro, 2000) and to mirror the period of human pregnancy and early life breastfeeding, as the final trimester of human pregnancy is equivalent to the first week of life in a murine mouse model, and the remaining week (postnatal days 8–14) during which the pups were exposed through their mother’s milk mirrors a postbirth breastfeeding period. The dose used was equivalent to the maximum recommended human dose of 80 mg/d (Maloney et al., 2018). Vehicle (VEH)-exposed dams received only 1% saccharin drinking water. Dams were randomly assigned to treatment groups.

Male and female adult mice (11–22 weeks of age), age-matched at imaging and from multiple independent litters of FLX and VEH dams, were used for imaging and processed in five independent cohorts (cohort 1: *n* = 7 FLX, 8 VEH; cohort 2: *n* = 8 FLX, 10 VEH; cohort 3: *n* = 9 FLX, 8 VEH; cohort 4: *n* = 9 FLX, 8 VEH; cohort 5: *n* = 17 FLX, 11 VEH). Both FLX and VEH groups included mice of both sexes (see Table 1 for number of males and females used in sex-specific analyses). Because this is a novel study without a prior basis for effect size, a power analysis using a standard estimated large effect size of 0.8 and with 80% power indicated a total sample size of 42 animals is required to reject the null hypothesis at the 0.05 α level. Mice were imaged during their 6 A.M. to 6 P.M. light cycle, with FLX and VEH mice counterbalanced across the imaging day. The health of the animals was monitored, and no differences between groups was observed. The experimenter was blinded to the treatment condition during data acquisition, processing, and preliminary analysis. Mice were excluded from analysis if movement of the animal suggesting wakefulness was detected, or from evoked response analysis if stimulation application failed and no change from baseline was recorded. The first two cohorts were wild type C57BL6/J (JAX:000664), while the later three were *Thy1*-GCaMP6f on the C57BL6/J background (JAX:024276). GCaMP6f expression allowed for population-based calcium recording from cortical excitatory neurons, in addition to concurrently acquired hemodynamics. All mice were fitted with a transparent Plexiglas window to facilitate imaging. Briefly, mice were sedated using isoflurane (3% induction, 1% maintenance, 0.5 l/min) with body temperature maintained at 37°C using a heating pad; the head was then shaved and the mouse prepared for surgery in a stereotactic restraint. An incision in the scalp was made along midline to expose the skull, and the skin was retracted to expose an ∼1-cm^2^ cortical field of view through the skull. A cranial window of Plexiglas was affixed to the dorsal surface of the intact skull using dental cement (C&B-Metabond, Parkell Inc.) which allowed for chronic, repeatable imaging.

**Table 1.** Statistical summary for Figures 1-Figures 4

Figures	Data structure	Type of test	*n*	Statistical significance
1*F*	Normal	Student’s *t* test	*n* = 48 FLX, 42 VEH	*p* > 0.05 with Bonferroni correction
1*J*	Normal	Student’s *t* test	*n* = 34 FLX, 25 VEH	*p* > 0.05 with Bonferroni correction
2*I*	Non-normal	Wilcoxon rank-sum test	*n* = 30 FLX, 24 VEH	Contralateral HbO: *p* = 0.13; ipsilateral HbO: *p* = 0.0046; Contralateral GCAMP6F: *p* = 0.060; ipsilateral GCAMP6F: *p* = 0.0011
2*I*	Non-normal	Wilcoxon rank-sum test	*n* = 18 FLX, 11 VEH (males)	Contralateral HbO: *p* = 0.14; ipsilateral HbO: *p* = 0.010; contralateral GCAMP6F: *p* = 0.06; ipsilateral GCAMP6F: *p* = 0.038
2*I*	Non-normal	Wilcoxon rank-sum test	*n* = 12 FLX, 13 VEH (females)	Contralateral HbO: *p* = 0.23; ipsilateral HbO: *p* = 0.13; contralateral GCAMP6F: *p* = 0.36; ipsilateral GCAMP6F: *p* = 0.012
3*D*	Non-normal	Wilcoxon rank-sum test	*n* = 30 FLX, 24 VEH	Contralateral HbO: *p* = 0.050; ipsilateral HbO: *p* = 0.043; contralateral GCAMP6F: *p* = 0.028; ipsilateral GCAMP6F: *p* = 0.014
3-1*A*,*C*	Non-normal	Wilcoxon rank-sum test	*n* = 18 FLX, 11 VEH (males)	Contralateral HbO: *p* = 0.031; ipsilateral HbO: *p* = 0.15; contralateral GCAMP6F: *p* = 0.034; ipsilateral GCAMP6F: *p* = 0.34
3-1*B*,*D*	Non-normal	Wilcoxon rank-sum test	*n* = 12 FLX, 13 VEH (females)	Contralateral HbO: *p* = 0.12; ipsilateral HbO: *p* = 0.075; contralateral GCAMP6F: *p* = 0.32; ipsilateral GCAMP6F: *p* = 0.0042
4*C*	Normal	Student’s *t* test	*n* = 30 FLX, 24 VEH	*p* < 0.05 for regions displayed

After a minimum of 48 h of postsurgery recovery, the mice were anesthetized for imaging by intraperitoneal injection of a ketamine/xylazine cocktail (86.9 mg/kg ketamine, 13.4 mg/kg xylazine, dosage 0.005 ml/kg) to avoid burst suppression (Ferron et al., 2009), and maintained at 37°C by heating pad (mTCII, Cell Microcontrols). Insertion of the mouse into the imaging apparatus was not initiated until full transition to the anesthetized state was observed, as measured by the absence of whisker movement or a response to a toe-pinch stimulus. The mouse’s head and imaging plane were secured using a platform mount which attached to and stabilized the Plexiglas window adhered to the mouse’s skull.

### Optical imaging system

Sequential illumination was provided at four wavelengths by a ring of light-emitting diodes (LEDS: 478, 588, 610, and 625 nm; RLS-5B475-S, B5B-4343-TY, B5B-435-30S, and OSCR5111A-WY, respectively; Roithner Lasertehnik) ∼10 cm above the mouse’s head, following the methods of White et al. (2011). Diffuse reflected light was captured by a cooled, frame-transfer EMCCD camera (iXon 897, Andor Technologies). Acquisition was synchronized with the LEDs’ sequential illumination and controlled via custom-written software (MATLAB, MathWorks) at a frame rate of 30 Hz per LED. The field of view was ∼1 cm^2^ with an anterior-posterior view from olfactory bulb to superior colliculus, consisting of 128 × 128 pixels with pixel size of ∼80 μm^2^. Cohorts 1 and 2 were imaged on this system.

### Optical/calcium fluorescence imaging system

**Figure 1.**
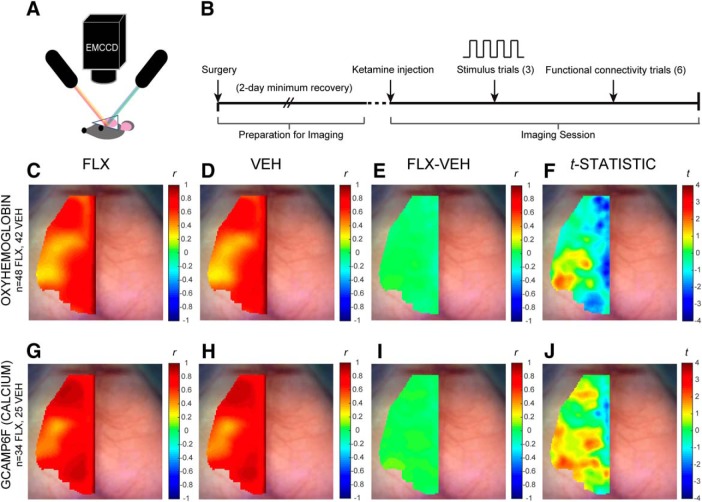
Resting-state homotopic contralateral functional connectivity in FLX mice does not significantly differ from VEH controls. ***A***, Optical fluorescence/OIS imaging system schematic: The cranial window lies directly below the camera as sequential illumination is delivered from light sources nearby. See Extended Data Figure 1-1 for a representative mouse brain image in which pixels not containing brain tissue are excluded from analysis and brain landmarks are identified. ***B***, Representation of OIS and evoked response imaging workflow. ***C–F***, Map of mean HbO_2_ homotopic contralateral functional connectivity (Pearson’s *r*) in (***C***) FLX (*n* = 48) and (***D***) VEH (*n* = 42) groups. ***E***, Mean difference between FLX and VEH ΔHbO_2_ maps (FLX-VEH). ***F***, *t* statistic map of FLX-VEH groups (uncorrected). ***G–J***, Map of mean GCaMP6f (ΔF) homotopic contralateral functional connectivity (Pearson’s *r*) in (***G***) FLX (*n* = 34) and (***H***) VEH (*n* = 25) groups. ***I***, Mean difference between FLX and VEH GCaMP6f maps (FLX-VEH). ***J***, *t* statistic map of FLX-VEH groups (uncorrected).

Sequential illumination and fluorophore excitation was provided by four LEDs situated ∼8 cm above the head [454 (GCaMP excitation), 523, 595, and 640 nm], following the methods of Wright et al. (2017; Fig. 1*A*). Diffuse reflected light and fluorescence emission was captured by a cooled, frame-transfer EMCCD camera (iXon 897, Andor Technologies). Acquisition was synchronized with the LEDs’ sequential illumination and controlled via custom-written software (MATLAB, MathWorks) at a frame rate of ∼16 Hz per LED. The field of view was ∼1 cm^2^ with an anterior-posterior view from olfactory bulb to superior colliculus consisting of an image plane sampled by 512 × 512 pixels, which are binned 4 × 4 down to 128 × 128 pixels with on-chip binning. The resulting pixel size was ∼80 μm^2^ on the mouse head. Cohorts 3–5 were imaged on this system.

10.1523/ENEURO.0238-19.2019.f1-1 Extended Data Figure 1-1 Image analysis is performed after excluding pixels not containing brain tissue and identifying landmarks to perform two-dimensional affine transformation. Representative mouse brain displaying mask excluding non-brain pixels within the 128 × 128-pixel field of view. Red denotes where the anterior suture between olfactory bulb and cerebrum intersects the midline. Blue denotes lambda, where superior colliculus and cerebrum intersect at midline. Download Figure 1-1, TIF file.

### Functional connectivity

Functional connectivity data under anesthesia and without stimulation (referred to here as resting state) was collected in each mouse for up to 30 min, split into six consecutive runs of 5 min (Fig. 1*B*). The first resting state run for each mouse was initiated approximately 3 min following any stimulus application from previous data collection blocks. Visual monitoring of the mouse was performed throughout, and on the detection of whisking or other movement indicating a return to wakefulness, the imaging session was terminated and the mouse was removed from the imaging system to complete its emergence from the anesthetized state.

### Forepaw stimulation

Electrical pulses were generated by an isolated pulse stimulator (Modell 2100, A-M Systems) and administered to the left forepaw by micro vascular clips (Roboz Surgical Instrument Co.). Stimulation data were collected in three consecutive runs of 5 min each. The block design was modeled on existing fMRI and functional optical imaging stimulation studies, in which mixed blocks of stimulation and resting state are common. Although some fMRI studies have shown a subtle alteration of resting state due to prior motor learning tasks (Albert et al., 2009; Zhang et al., 2014), these effects have not been explored as extensively with mice and optical intrinsic signal (OIS). In either case, in this study we used the same design structure for both groups of mice and thus the effect or lack of effect would equally influence both groups. The 5-min run consisted of five 1-min blocks; each with an initial 5-s rest period, followed by 10 s of electrical stimulation (frequency: 3 Hz, pulse duration: 300 μs, current: 0.75 mA), and 45 s of rest (Fig. 1*A*). The five 1-min blocks were later block averaged for each stimulus trial.

### Imaging data processing and analysis

Data were processed using MATLAB and an imaging analysis pipeline following that previously described in the literature (Bauer et al., 2014; Wright et al., 2017) with modifications as described. For calcium imaging, approximated absorption coefficients for GCaMP6 excitation and emission were calculated and used to correct for absorption of GCaMP6 fluorescence by hemodynamic fluctuations, as described elsewhere (Ma et al., 2016). For all cohorts, a binary brain mask was created by delineating brain borders in the field of view using the roipoly function in MATLAB. Following a previously published approach (White et al., 2011), the co-registration processing began with defining an individual-specific binary brain mask that was created for each mouse and used to eliminate pixels not containing brain tissue (Extended Data Fig. 1-1). Within the mouse images, we manually labeled the locations of lambda, where superior colliculus and cerebrum intersect at midline, and the anterior suture between the olfactory bulb and cerebrum at midline. These coordinates were then used to co-register all mice to common Paxinos atlas space (Franklin and Paxinos, 2008) using a two-dimensional affine transformation. Runs with light levels with >1% variance throughout the time series were discarded. In addition to visual inspection, movement of the animal was tracked by calculating pixel displacement for each run, and runs with detected movement were subsequently discarded. The runs which passed these quality-control steps were used to create average functional connectivity maps or stimulus time traces for each mouse. After quality control, for resting state analysis an average of 70% of the data per mouse was used, with a minimum requirement of 5 min of data from each mouse. For stimulation an average of 80% of the data per mouse was used, with a minimum of 5 min (five stimulation presentations) of data from each mouse.

The processed resting-state functional connectivity and evoked-response data produced were used to analyze homotopic contralateral function connectivity (HCFC) and change from baseline oxyhemoglobin (ΔHbO_2_) or fluorescence (ΔF) levels across time, respectively. Resting-state analyses included hemodynamic data from cohorts 1–5 to maximize sample size, and calcium data from the GCaMP-positive cohorts, cohorts 3–5. The forepaw stimulation analyses used cohorts 1 and 2 to define regions of interests (ROIs), which were then used to subsequently analyze the hemodynamic and calcium response data from cohorts 3–5. Figure 1*C–F* includes more mice than Figure 1*G–J* because Figure 1*C–F* has hemodynamic measures and includes the data of cohorts 1–5 to include the maximum sample size. Figure 1*G–J* includes less mice because it is a calcium measure and cohorts 1–2 were GCaMP-negative and therefore had no fluorophore excitation to record and average into the functional connectivity maps.

Hemodynamic data were filtered to the 0.009- to 0.25-Hz band (stimulation data) or 0.009- to 0.08-Hz band (resting-state data), whereas calcium signal was filtered to the 0.1- to 6.0-Hz band (stimulation data) or 0.4- to 4.0-Hz band (resting-state data). Global signal regression was performed on all data to remove sources of variance, except where indicated otherwise. An averaged map of pixel-by-pixel comparisons of HCFC for each mouse was produced by use of the brain mask and atlas-based midline coordinates. Because the homotopic contralateral connectivity methods compare pairs of pixels that are reflections of each other between the left and right hemisphere, the left and right hemisphere maps are mirror images about midline. We therefore report via heat map the connectivity coefficients in the left hemisphere and display the dorsal cortical surface and its landmarks on the right hemisphere, to provide anatomic context when viewing the values displayed across midline.

Forepaw stimulation data were analyzed both across time and at stimulus end (the mean of 11 frames surrounding *t* = 10 s). Each stimulus block was normalized to mean baseline hemoglobin or calcium levels in the 5 s before stimulus onset. To calculate change in hemoglobin or calcium levels across time in cohorts 3–5, two ROIs were defined using the independent *t* statistic map derived from cohorts 1 and 2’s FLX-VEH differences at stimulus end (Extended Data Fig. 2-1). A circle (*r* = 15 pixels or ∼1.2 mm) centered on the maximum HbO_2_ value in stimulated right (contralateral) cortex was drawn to produce one ROI, then reflected across midline to create a homotopic ROI in left (ipsilateral) cortex. All half-maximum *t* statistic pixels in the contralateral region and half-minimum *t* statistic pixels in ipsilateral region were identified (Extended Data Fig. 2-1*D*), and the time traces of these pixels within each of the two regions (contralateral and ipsilateral) were averaged and plotted across time. Pearson *r* values were Fisher *z*-transformed before all mathematical operations.

**Figure 2.**
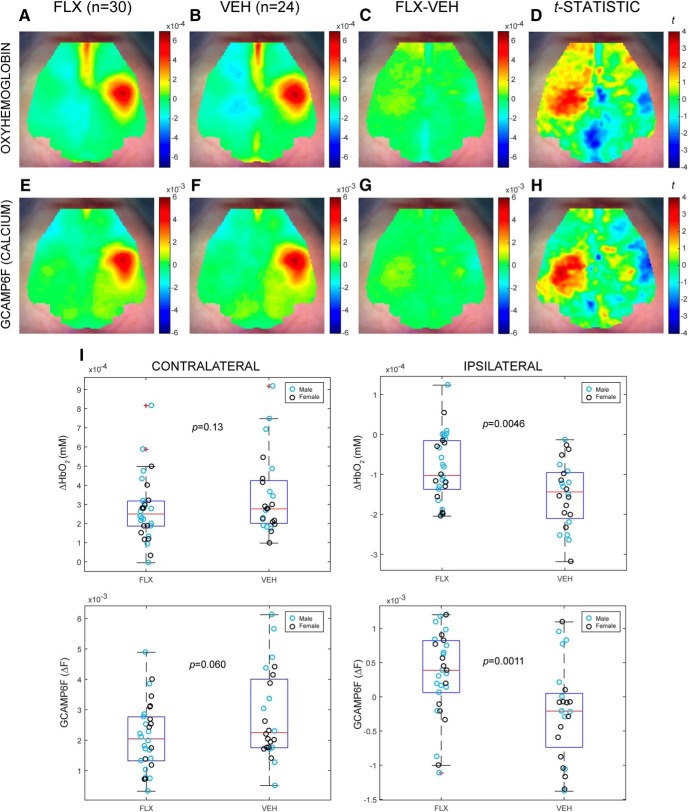
Mean HbO_2_ and calcium response to left forepaw stimulation at *t* = 10 s is decreased in FLX contralateral somatosensory cortex and increased in ipsilateral cortex. ***A***, ***B***, Map of mean HbO_2_ (mM) in (***A***) FLX (*n* = 30) and (***B***) VEH (*n* = 24) groups. See Extended Data Figure 2-2 for the mean HbO_2_ activation map overlaid with cortical region boundaries as defined by the Paxinos atlas. ***C***, Mean difference between FLX and VEH HbO_2_ maps (FLX-VEH). ***D***, *t* statistic map of FLX-VEH HbO_2_ (uncorrected). ***E***, ***F***, Map of mean GCaMP6f calcium signal (ΔF) in (***E***) FLX (*n* = 30) and (***F***) VEH (*n* = 24) groups. ***G***, Mean difference between FLX and VEH calcium maps (FLX-VEH). ***H***, *t* statistic map of FLX-VEH calcium groups (uncorrected). ***I***, Boxplots and scatterplots of HbO_2_ (first row) and GCaMP6f (second row) changes from baseline at *t* = 10 s in contralateral (left) and ipsilateral (right) ROIs defined by the independent dataset of cohorts 1 and 2 (see Extended Data Fig. 2-1 for ROI boundaries and the maps from cohorts 1 and 2 by which the ROIs are defined). Sex of individuals within FLX and VEH groups indicated by blue (males) and black (females).

10.1523/ENEURO.0238-19.2019.f2-1 Extended Data Figure 2-1 Contralateral and ipsilateral ROIs defined by half-maximum or half-minimum pixels from the *t* statistic map of GCaMP-negative cohorts 1 and 2. ***A***, Mean HbO_2_ of the GCaMP6f-negative (cohorts 1 and 2) mice at end of forepaw stimulation (*t* = 10 s; FLX *n* = 13, VEH *n* = 17). Grey circles delineate the regions of radius 15 pixels from which half-minimum *t* statistic pixels were selected for the contralateral ROI (black region) and half-maximum *t* statistic pixels were selected for the ipsilateral ROI (blue region). ***B***, Mean HbO_2_ of the GCaMP6f-positive cohorts (cohorts 3–5) at end of forepaw stimulation (*t* = 10 s) with predefined ROIs overlaid (FLX *n* = 30, VEH *n* = 24). ***C***, Mean GCaMP6f calcium (ΔF) of the GCaMP6f-positive cohorts (cohorts 3–5) at end of forepaw stimulation (*t* = 10 s) with predefined ROIs overlaid (FLX *n* = 30, VEH *n* = 24). ***D***, HbO_2_ *t* statistic map of the GCaMP6f-negative (cohorts 1 and 2) mice at end of forepaw stimulation (*t* = 10 s; FLX *n* = 13, VEH *n* = 17). Grey circles delineate the regions of radius 15 pixels from which half-minimum *t* statistic pixels were selected for the contralateral ROI (black region) and half-maximum *t* statistic pixels were selected for the ipsilateral ROI (blue region). ***E***, HbO_2_ *t* statistic map of the GCaMP6f-positive cohorts (cohorts 3–5) at end of forepaw stimulation (*t* = 10 s; FLX *n* = 30, VEH *n* = 24). ***F***, GCaMP6f calcium *t* statistic map of the GCaMP6f-positive cohorts (cohorts 3–5) at end of forepaw stimulation (*t* = 10 s; FLX *n* = 30, VEH *n* = 24). Download Figure 2-1, TIF file.

### Statistical analysis

All statistical comparisons were performed using MATLAB v9.2 (MathWorks). Statistical analyses were performed to determine if there was a significant difference between FLX and VEH groups in stimulation and resting-state trials. Statistical significance was determined by two-tailed unpaired Student’s *t* tests in all instances except where otherwise stated. For HCFC analyses, a *t* test was performed for each brain pixel pair and represented by a heat map value in that pixel within the cortical image. With the data smoothed by a Gaussian blur with full width at half maximum of 0.096 mm, and a field of view of 22.9 mm^2^, an estimated 792 independent measures exist. The minimum uncorrected *p* value is 0.0015 (HbO_2_) or 6.85E-4 (Ca^2+^), but with a Bonferroni correction, performed for 792 tests, the corrected *p* value threshold is 6E-5, and therefore none of the comparisons survive correction as they are at least an order of magnitude too high. A one-tailed Wilcoxon rank-sum test based on the direction of effect from cohorts 1 and 2’s FLX-VEH differences was used to assess differences in mean signal in the Contralateral and Ipsilateral ROIs at end of stimulation. A one-tailed Wilcoxon rank-sum test was also used to assess differences in area under the curve (AUC) from the onset to end of stimulation between FLX and VEH groups. All pixel-by-pixel comparisons were Bonferroni corrected unless otherwise stated. A critical α of 0.05 was used for all tests to determine significance.

## Results

### Resting-state hemodynamics remain unaltered following maternal FLX exposure

To determine whether transient developmental FLX exposure can result in long-term alterations in functional connectivity observable in adulthood, we examined FLX-exposed mice for changes in resting-state functional connectivity using an OIS imaging system. We assessed cortical activity in ∼45 mice per condition, across five cohorts, using both direct measures of neuronal activity (Ca^2+^ imaging, cohorts 3–5) as well as hemodynamics (cohorts 1–5), representing the kind of indirect assessment that would be available in humans.

In the absence of a task or applied stimulus, correlation between individual cortical regions is used to operationally define resting-state functional connectivity independent of structural connectivity (Biswal et al., 1995). Specifically, functional connectivity between corresponding regions across hemispheres is high because of the regions’ similar functions, as exemplified by the correlation between left and right motor areas. This homotopic contralateral functional connectivity (HCFC) can be assessed to identify disruption in disease models (Bauer et al., 2014). Comparison of HCFC between FLX and VEH groups (Fig. 1*C–F*) identified no significant alterations in the cortex: The HbO_2_ HCFC patterns in FLX (Fig. 1*C*) and VEH (Fig. 1*D*) groups did not significantly diverge from each other in the HbO_2_ signal (*t* test, *p* > 0.05 with Bonferroni correction; Fig. 1*E*,*F*). The GCaMP6f calcium data collected simultaneously with the hemodynamic data in cohorts 3–5 also showed a lack of significant difference (Fig. 1*I*,*J*) between FLX (Fig. 1*G*) and VEH (Fig. 1*H*) HCFC maps (*t* test, *p* > 0.05 with Bonferroni correction). This finding confirmed that the similarity in FLX and VEH resting-state HCFC patterns seen in HbO_2_ were also reflected in calcium. Developmental FLX exposure therefore did not significantly alter resting-state HCFC as assessed by either hemodynamics or a more direct calcium-based measure of neural activity.

### Developmental FLX exposure alters stimulus-induced cortical hemodynamics and calcium activity

Sensory aberrations such as altered behavioral responses to pain have previously been reported in neonates with prenatal SSRI exposure and the critical, transient 5HT role in the development of thalamocortical projections suggests that perception or management of sensory stimuli may be altered in individuals with perinatal FLX exposure (McCormick and Bal, 1994; Lebrand et al., 1996; Allen et al., 2017). To probe whether developmental FLX exposure alters sensory responsiveness to environmental stimuli, we therefore examined the evoked cortical response to a somatosensory stimulus. Left forepaw stimulation was applied to FLX and VEH mice in a block design, and changes in HbO_2_ and calcium signal were measured cortex-wide. The stimulation data of the GCaMP6f-negative cohorts 1 and 2 (Extended Data Fig. 2-1) were used to define ROIs that were subsequently used to evaluate cohorts 3–5’s HbO_2_ and calcium responses (Fig. 2). As expected, the observed region of greatest activation in all groups was the forepaw region of the somatosensory cortex, as defined by the cortical regions in the Paxinos histologic atlas (Franklin and Paxinos, 2008) and adapted to be a dorsal-view cortical map in White et al. (2011; Extended Data Fig. 2-2). HbO_2_ response at the end of the 10-s stimulation block (*t* = 10 s) identified ROIs in both the contralateral and ipsilateral cortices, with the FLX group (Fig. 2*A*) displaying decreased HbO_2_ amplitude compared to the VEH controls (Fig. 2*B*) in the contralateral region focused over the stimulated somatosensory cortex (Fig. 2*C*,*D*). A comparative increase of HbO_2_ in ipsilateral cortex in FLX as compared to VEH is also evident at end of stimulation (Fig. 2*D*). As HbO_2_ is only an indirect measure of neural activity, this result could reflect either changes in neural activity or in hemodynamics. Therefore, we also examined calcium transients in our GCaMP6f cohorts. This decrease in contralateral activation and comparative increase in ipsilateral cortical activity during and immediately following stimulation was observed in the three GCaMP6f cohorts’ calcium dynamics as well (Fig. 2*E–H*), confirming that underlying neural activity is altered on somatosensory stimulation. Contralateral and ipsilateral ROIs for analysis of cohorts 3–5 were defined by GCaMP-negative cohort 1 and 2’s mean HbO_2_ maps, which displayed an increase in ipsilateral signal and decrease in contralateral signal within these ROIs in FLX compared to VEH (Extended Data Fig. 2-1). Similarly, cohorts 3–5 displayed an increase in ipsilateral ROI signal in both contrasts (Wilcoxon rank-sum test; HbO_2_ *p* = 0.0046, Ca^2+^ *p* = 0.0011), although the contralateral ROI’s decrease in both HbO_2_ and calcium signal was not significant (Wilcoxon rank-sum test; HbO_2_ *p* = 0.13, Ca^2+^ *p* = 0.060; Fig. 2*I*). When animals of each sex were analyzed separately, the same significant increase in ipsilateral ROI signal was observed in males’ HbO_2_ and calcium response as well as females’ calcium signal (Wilcoxon rank-sum test; males HbO_2_ *p* = 0.010, Ca^2+^ *p* = 0.038, females HbO_2_ *p* = 0.13, Ca^2+^ *p* = 0.012; Table 1). The reduction in HbO_2_ and calcium absolute signal amplitude in the ipsilateral response typically observed in VEH controls therefore demonstrates that developmental FLX exposure alters the strength of the cortical activity evoked by somatosensory stimulation.

10.1523/ENEURO.0238-19.2019.f2-2 Extended Data Figure 2-2 Left forepaw stimulation results in activation of the right cortical forepaw region, as defined by the Paxinos atlas. Mean HbO_2_ stimulation averaged across all mice and groups with forepaw (black), M1 (red), hindpaw (blue), and whisker barrel (magenta) functional region boundaries mapped on the mouse cortex. Download Figure 2-2, TIF file.

### Developmental FLX exposure decreases cortical activation contralateral to the stimulated paw and reduces ipsilateral inhibition

**Figure 3.**
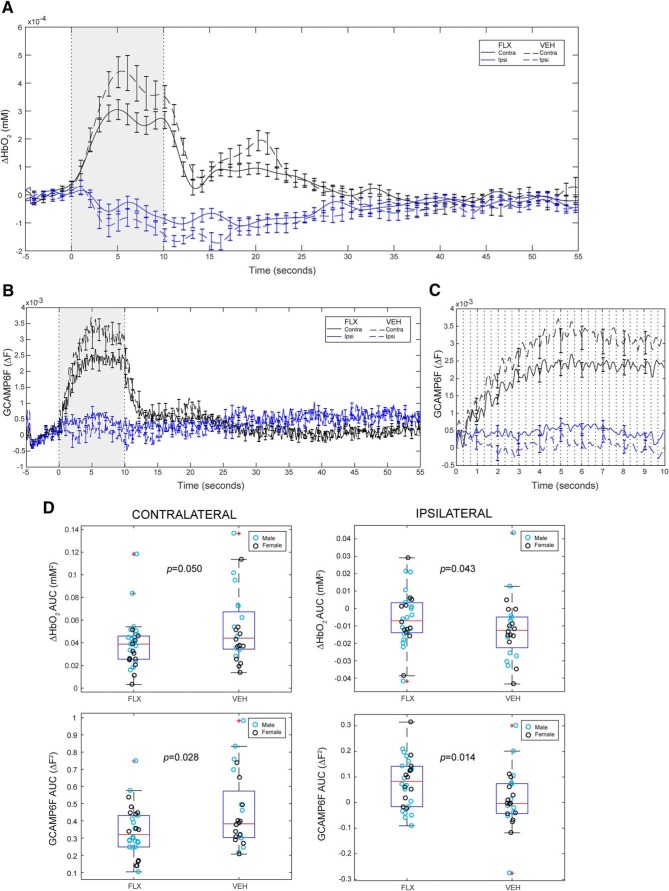
HbO_2_ and Ca^2+^ response amplitude is decreased in ROI contralateral to stimulated forepaw and altered in ipsilateral ROI. ***A***, Block-averaged time trace of mean ΔHbO_2_ response to forepaw stimulation in each ROI (FLX *n* = 30, VEH *n* = 24; black = contralateral, blue = ipsilateral). Left forepaw stimulation at 3 Hz applied *t* = 0 s to *t* = 10 s. ***B***, Block-averaged time trace of GCaMP6 Ca^2+^ response to forepaw stimulation in each ROI (FLX *n* = 30, VEH *n* = 24; black = contralateral, blue = ipsilateral). Left forepaw stimulation at 3 Hz applied *t* = 0 s to *t* = 10 s. ***C***, Detail of block-averaged time trace of mean GCaMP6 Ca^2+^ response to forepaw stimulation during time of stimulation (*t* = 0–10 s; FLX *n* = 30, VEH *n* = 24). Vertical black lines represent timing of 3-Hz stimulus presentation. ***D***, Boxplots and scatterplots of HbO_2_ (first row) and GCaMP6f (second row) AUC for contralateral (left) and ipsilateral (right) cortical ROIs during stimulation (*t* = 0–10 s). Sex of individuals within FLX and VEH groups indicated by blue (males) and black (females). See Extended Data Fig. 3-1 for block-averaged time traces separated by sex.

The analysis above defined regions that at a single time point, following stimulation, show differences between FLX and VEH groups in multiple cortical regions. We therefore reanalyzed the data from cohorts 3–5 to determine whether this effect is consistent across time. Mean HbO_2_ and calcium activity of the two ROIs (contralateral and ipsilateral to the stimulated paw) were traced across the 60-s stimulation block. These regions were defined relative to the stimulated paw and included contralateral and ipsilateral ROIs (Extended Data Fig. 2-1). FLX mice displayed a mean decrease in HbO_2_ signal amplitude compared to the VEH group in the contralateral ROI during the 10-s stimulation block (Fig. 3*A*). As the end of stimulation (*t* = 10 s) approached, absolute HbO_2_ signal amplitude in the ipsilateral ROI also decreased in the FLX group as compared to VEH (Fig. 3*A*). HbO_2_ AUC from the onset to end of stimulation for the contralateral and ipsilateral ROIs were both significantly different in the FLX mice compared to VEH (Wilcoxon rank-sum test; contralateral AUC *p* = 0.050, ipsilateral AUC *p* = 0.043; Fig. 3*D*). This AUC finding was replicated in the calcium signal, which displayed significant differences in both ROIs (Wilcoxon rank-sum test; contralateral AUC *p* = 0.028, ipsilateral AUC *p* = 0.014; Fig. 3*B–D*). Although not deliberately powered for analysis by sex, when analyzed separately, males displayed a significant decrease in both the HbO_2_ and calcium contralateral response magnitude (AUC of the contralateral ROI response), while females did not display a significant decrease in HbO_2_ contralateral response magnitude (contralateral ROI AUC in the HbO_2_ signal, Wilcoxon rank-sum test; males HbO_2_ contralateral AUC *p* = 0.031, Ca^2+^ *p* = 0.034, females contralateral HbO_2_ AUC *p* = 0.12, Ca^2+^ *p* = 0.32; Extended Data Fig. 3-1; Table 1). In the ipsilateral ROI response, FLX and VEH groups analyzed as distinct male or female populations were not significantly different but females displayed a trend toward increased ipsilateral ROI AUC in the HbO_2_ signal and demonstrated a significant increase in ipsilateral AUC response in the calcium signal (Wilcoxon rank-sum test; males HbO_2_ ipsilateral AUC *p* = 0.15, Ca^2+^ *p* = 0.34, females HbO_2_ ipsilateral AUC *p* = 0.075, Ca^2+^ *p* = 0.0042; Extended Data Fig. 3-1; Table 1). These results confirmed that the differences in signal intensity observed at the end of the 10-s stimulation block were consistently observed across time during the application of the somatosensory stimulus. Of particular note, the calcium and hemodynamic signals display the same gross difference across time and suggest that HbO_2_ is indeed reflecting an underlying neural deficit.

10.1523/ENEURO.0238-19.2019.f3-1 Extended Data Figure 3-1 HbO2 and Ca^2+^ response amplitude is altered in both males and female FLX mice during forepaw stimulation. ***A***, Block-averaged time trace of mean ΔHbO_2_ response in males to forepaw stimulation in each ROI (FLX *n* = 18, VEH *n* = 11; black = contralateral, blue = ipsilateral). Left forepaw stimulation at 3 Hz applied *t* = 0 s to *t* = 10 s. ***B***, Block-averaged time trace of mean ΔHbO_2_ response in females to forepaw stimulation in each ROI (FLX *n* = 12, VEH *n* = 13; black = contralateral, blue = ipsilateral). Left forepaw stimulation at 3 Hz applied *t* = 0 s to *t* = 10 s. ***C***, Block-averaged time trace of GCaMP6 Ca^2+^ response in males to forepaw stimulation in each ROI (FLX *n* = 18, VEH *n* = 11; black = contralateral, blue = ipsilateral). Left forepaw stimulation at 3 Hz applied *t* = 0 s to *t* = 10 s. ***D***, Block-averaged time trace of GCaMP6 Ca^2+^ response in females to forepaw stimulation in each ROI (FLX *n* = 12, VEH *n* = 13; black = contralateral, blue = ipsilateral). Left forepaw stimulation at 3 Hz applied *t* = 0 s to *t* = 10 s. Download Figure 3-1, TIF file.

### Abnormalities in HbO_2_ and calcium evoked response co-localize in the cortex

**Figure 4.**
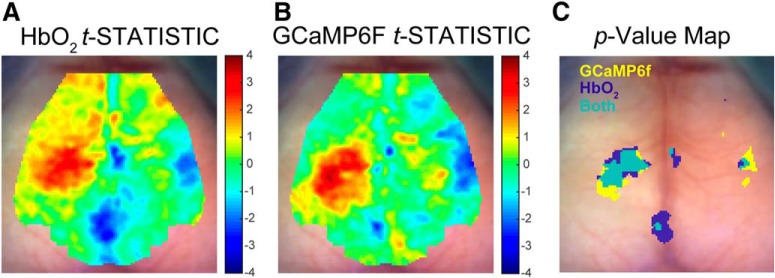
Increases in FLX mice’s HbO_2_ and calcium response co-localize in cortex ipsilateral to forepaw stimulation. ***A***, HbO_2_ *t* statistic map of FLX-VEH (uncorrected; FLX *n* = 30, VEH *n* = 24). ***B***, GCaMP6f *t* statistic map of FLX-VEH (uncorrected; FLX *n* = 30, VEH *n* = 24). ***C***, Overlay figure of HbO_2_ and GCaMP6f uncorrected *p* < 0.05 maps (blue = HbO_2_, yellow = GCaMP6f, green = both).

While the calcium and hemodynamic signals both differed significantly in the same direction within the two ROIs described, it was unclear whether abnormalities in HbO_2_ and GCaMP6f would occur together in other cortical areas. We therefore evaluated if the calcium and hemodynamic signals spatially co-localized in areas outside of the two ROIs by overlaying binary cortical maps for each contrast that represented each pixel where there was a significant FLX-VEH difference (*p* < 0.05, uncorrected; Fig. 4). HbO_2_ and calcium displayed an overlapping area of strong FLX-VEH difference in contralateral cortex and a co-localization region in ipsilateral cortex of ∼1.5 mm^2^. This suggests that the two contrasts overall show similar activation patterns in response to somatosensory stimulation.

## Discussion

Overall, the changes observed in evoked response but not functional connectivity in this mouse model of perinatal or developmental SSRI exposure suggest that sensory information processing is more strongly affected by this environmental influence than resting state functional connectivity is. The lack of significant difference in homotopic contralateral functional connectivity that we found suggests that the correlation structure in baseline hemodynamic and neuronal dynamics are largely undisturbed in the FLX mouse cortex. This lack of significant result may also reflect, however, the severity or variability in phenotype that results from perinatal SSRI exposure. With a relatively large sample size of ∼40 mice per group, it would be reasonable to expect that even a relatively small effect would be identified. However, perinatal SSRI exposure in humans, if it does contribute to risk beyond maternal diagnosis, has a modest effect size and thus does not lead to a diagnosis of psychiatric illness or other neurodevelopmental conditions in a high percentage of cases (Malm et al., 2016; Gingrich et al., 2017). If mice also show this variable penetrance, then future studies might leverage a behavioral variable or other metric to stratify data for further analysis.

In contrast to the resting state analysis, the abnormalities observed in cortical response to forepaw stimulation showed a reliable effect, detectable across three cohorts. This suggests that perinatal SSRI exposure alters sensory processing or gating in a way that has lasting repercussions observable even in adulthood. The reduced response in cortex contralateral to the stimulated paw as well as reduced negative ipsilateral signal during stimulation trials indicates that somatosensory inputs are abnormally received or processed in the cortex. The abnormal cortical response in ipsilateral cortex suggests reduced inhibition, which may be caused by thalamocortical dysfunction linked to morphologic abnormalities previously reported in the literature from SSRI exposure (Lee and Lee, 2012). Suppression of activity in ipsilateral somatosensory cortex during stimulus presentation plays a role in the successful execution of sensory gating (Staines et al., 2002) and, by extension, the ability to process and react to sensory stimuli in an organism’s environment as needed. Abnormal ipsilateral cortical inhibition suggests that developmental SSRI exposure causes long-term sensory gating and processing abnormalities. The left forepaw was exclusively stimulated in these assays, but similar effects would be expected if right forepaw was stimulated instead, due to the equal number of left-handed and right-handed individuals in the C57Bl/6J population (Collins, 1968).

Finally, the abnormalities in cortical response following perinatal SSRI exposure we detected suggest that HbO_2_ and GCaMP calcium dynamics during somatosensory stimulation are coupled temporally and spatially across the cortex. During stimulation, both contrasts displayed a loss of signal amplitude in contralateral ROI and abnormalities in ipsilateral cortex. The cortical regions with the largest FLX-VEH differences in HbO_2_ and calcium also displayed significant overlap at the end of stimulation. This suggests that altered neurovascular coupling is not the immediate underlying cause for the HbO_2_ deficits we observe following perinatal SSRI exposure. Future studies might leverage this finding as added confirmation that hemodynamic methods or blood oxygen level-dependent methods in humans are a valid way to examine neural activity in 5HT dysfunction or following early SSRI exposure in humans or mammalian experimental systems.

This abnormal cortical response to somatosensory stimulation following perinatal FLX exposure that we have described here appears to reflect and further characterize the same underlying phenomenon as other deficits reported in the literature. Early SSRI exposure in rodents has previously been linked to long-term morphologic changes to both the somatosensory cortex and thalamocortical afferents to the region (Lee, 2009; Smit-Rigter et al., 2012). Alterations in somatosensory-related behaviors have also been observed (Lee, 2009; Maloney et al., 2018), but behavior phenotypes are complex and difficult to directly link to neural alterations. This functional neuroimaging phenotype, which suggests sensory gating or processing abnormalities on a circuit level, helps bridge the gap between morphologic and behavioral effects of perinatal SSRI exposure.

We used both sexes of animals so that our results would be more generalizable, and although our study was not deliberately powered for analysis by sex, *post hoc* the trends seen in the evoked response to forepaw stimulation suggest that female and male brains respond similarly to perinatal SSRI exposure. It is true that in both the hemodynamic and calcium measures, differences in males’ contralateral response and females’ ipsilateral response appeared more prominent, suggesting that further studies focused on sex differences could be a promising research direction. Limitations of this study also provide avenues for further research into the effects of perinatal SSRI exposure. Although necessary for our forepaw stimulation paradigm, imaging mice under the effects of anesthesia may introduce confounds to neuroimaging results (Wright et al., 2017), and therefore a study of resting-state dynamics or evoked response in wakefulness using visual or whisker stimulation (Berwick et al., 2002; Martin et al., 2013; Carandini et al., 2015; Xiao et al., 2017) could identify functional neurophenotypes that we were underpowered to observe. In addition, we documented the effects of perinatal SSRI exposure in adulthood, but performing similar neuroimaging assays at earlier timepoints during development (Kozberg et al., 2016) would help elucidate the developmental trajectory of these functional abnormalities and potentially serve as a biomarker to determine what portion of the exposed population may develop long-term deficits. Comparison of individuals’ somatosensory stimulation response in the cortex with their performance on tactile behavioral assays such as the von Frey test of somatosensation should also be considered, as it has previously been shown to be sensitive to developmental SSRI exposure perturbations (Maloney et al., 2018), and has the potential to provide insights into individual variability in populations developmentally exposed to SSRIs. In future studies it will be important to consider using functional MRI in mice, to evaluate whether these OIS hemodynamic findings are replicated using fMRI’s BOLD signal and potentially explore subcortical elements of the circuit in which the abnormal evoked somatosensory response was observed. Future functional neuroimaging studies in awake mice or at earlier stages in development would provide important information on the long-term effects of perinatal SSRI exposure or other genetic and environmental perturbations affecting neurodevelopment.

Taken together, our findings provide evidence that perinatal SSRI exposure causes long-term functional deficits observable in the brain in adulthood. While we did not observe changes in resting-state dynamics between homotopic contralateral regions, cortical response to somatosensory stimulation was altered in FLX mice. The observed altered responses in both cortical hemispheres after perinatal exposure to an SSRI suggest that the evoked cortical response is both diminished in magnitude and slightly shifted spatially compared to the response in the healthy brain, and bridges the morphologic and behavioral phenotypic abnormalities previously seen in models of early SSRI exposure.

## Synthesis

Reviewing Editor: Cheryl Sisk, Michigan State University Decisions are customarily a result of the Reviewing Editor and the peer reviewers coming together and discussing their recommendations until a consensus is reached. When revisions are invited, a fact-based synthesis statement explaining their decision and outlining what is needed to prepare a revision will be listed below. The following reviewer(s) agreed to reveal their identity: Keerthi Krishnan.

The manuscript seeks to characterize the long-term maternal SSRI exposure during pre- and postnatal development effects on processing tactile stimulation. The premise of the study has implications on the role of maternal SSRI usage in human neurodevelopment and connectivity. Reviewers raised a number of methodological questions and also recommend clarifications on issues regarding meaningful data analysis and critical synthesis of the implications of the findings to help reach a wider audience.

Experimental design, methods, and data analysis

1. The authors are commended for using a robust sample size for experimentation and analysis. However, though both sexes are used, there is no mention of analysis by sex. Might this contribute to the variability in the analysis? Do female and male brains respond similarly to SSRI treatment? Is this why they were grouped together?

2. Clarification on why this study design included both pre- and post-natal SSRI treatment would be helpful.

3. Five cohorts were acquired by using two different settings. Did authors consider the signal difference between cohorts 1-2 and 3-5 because all of mice of five cohorts were directly used in the group analysis?

4. Figure 1B shows that forepaw stimulation of 15 minutes was conducted before the resting state recording of 30 minutes. In general, in order to avoid effects of the task stimulation, the rest state scan precedes all of task scans. It is not clear why the resting state measurement was not acquired before the electrical stimulation in the present study. Could authors supply a brief explanation?

5. The intervals of the ketamine injection to forepaw stimulation and forepaw stimulation to resting state were not mentioned in either the Figure 1 legend or the methods section. Are these intervals of each mouse same? Particularly, is the interval of the forepaw stimulation to resting state long enough to eliminate the effects of stimulations for the resting state functional connectivity?

6. Was the binary brain mask created for each mouse before the co-registration processing? Could authors add an example figure to show the boundary of the binary brain mask and corresponding landmarks?

7. Authors need to add more details about the homotopic contralateral functional connectivity approach. It is not clear why Figure C-J only shows the averaged functional connectivity map of the left hemisphere. Was the contralateral functional connectivity of each pixel calculated in this analysis? Could authors also show the averaged map of the right hemisphere as shown in Figures 2 and 2-1?

8. Authors need to add more detail of the Bonferroni correction approach. What are the uncorrected p values and the amount of the one-tailed Wilcoxon rank sum or student test in the calculation of Bonferroni correction?

9. Data from five independent cohorts (Cohort 1: n=7 FLX, 8 VEH; Cohort 2: n=8,10; Cohort 3: n=9,8; Cohort 4: n=9,8; Cohort 5: n=17,11, total 50 FLX and 45 VEH) were acquired in the present study. However, data from a fewer number of mice of are reported. Authors should add a detailed rationale for why some mice were discarded for each analysis. For example, in Figure 1, 48 FLX and 42 VEH mice were used in C-F, and 34 FLX and 25 VEH mice were used in G-J.

10. 15 minutes and 30 minutes are acquired for the forepaw stimulation and the resting state recording, respectively, for each mouse. Could authors also supply a brief summary about how many minutes' data were used after the quality control and pre-processing procedure?

11. The authors state on page 14, that “as expected, the observed region of greatest activation was the forepaw region of the somatosensory cortex”. Please show data for the levels of activation. How does this compare with other SS1 regions, such as the barrel field? Is there minimal activation in other SS1 regions over forepaw?

Discussion and interpretation

12. It is unclear what “a decrease in contralateral activation and comparative increase in ipsilateral activity means”, as there were no changes in baseline. What are the speculated consequences of this result? Could this be due to handedness of the mice being compared? Were the mice tested for this before the imaging or forepaw stimulation?

13. Left forepaw was stimulated in these assays. Would the authors expect to observe similar, reverse results if the right paw was stimulated? This could point at the level of activation vs. morphological/connectivity issue discussed in the paper.

14. Reviewers recommend analyzing contralateral and ipsilateral imaging data per individual mouse to determine if these results might have consequences to individual mice in processing tactile information - which is one of the points the authors suggest for implications in variability in humans.

15. fMRI is very powerful non-invasive imaging approach, which can complement the invasive 3D BOLD data for resting state functional connectivity and forepaw stimulation approach. Could authors add some discussion about the fMRI and optical imaging approach? Will authors plan to run further fMRI studies in the future?

Minor

16. Pg.24: “gaiting” was mentioned a few times. Is “gating” the intended word?

## Author Response

Response to reviews, MS No. eN-NWR-0238-19

Reviewer & Editor Comments:

Computational Neuroscience Model Code Accessibility Comments for Author (Required):

N/A

Significance Statement Comments for Author (Required):

N/A

Comments on the Visual Abstract for Author (Required):

N/A

Synthesis Statement for Author (Required):

“The manuscript seeks to characterize the long-term maternal SSRI exposure during pre- and postnatal development effects on processing tactile stimulation. The premise of the study has implications on the role of maternal SSRI usage in human neurodevelopment and connectivity. Reviewers raised a number of methodological questions and also recommend clarifications on issues regarding meaningful data analysis and critical synthesis of the implications of the findings to help reach a wider audience.

Experimental design, methods, and data analysis

1. The authors are commended for using a robust sample size for experimentation and analysis. However, though both sexes are used, there is no mention of analysis by sex. Might this contribute to the variability in the analysis? Do female and male brains respond similarly to SSRI treatment? Is this why they were grouped together?

We thank the reviewers for bringing up this point regarding sex differences and their potential contribution to variability in the analysis. While the project was not designed or powered to discriminate differences in response between sexes, data were collected in both males and females. In general both sexes responded similarly. We have added the following to the paper:

1. Color coding by sex of the scatter plot data in Figure 2 and 3 and the following text regarding Figure 2I's data in the text:

a. “When animals of each sex were analyzed separately, the same significant increase in ipsilateral ROI signal was observed in males' HbO2 and calcium response as well as females' calcium signal (Wilcoxon rank sum test; males HbO2 p=0.010, Ca2+ p=0.038, females HbO2 p=0.13, Ca2+ p=0.012; Table 1).”

2. Evoked response data in Extended Figure 3 separated by sex and the following text in the Results:

a. “Although not deliberately powered for analysis by sex, when analyzed separately, males displayed a significant decrease in both the HbO2 and calcium contralateral response magnitude (AUC of the contralateral ROI response), while females did not display a significant decrease in HbO2 contralateral response magnitude (contralateral ROI AUC in the HbO2 signal, Wilcoxon rank sum test; males HbO2 contralateral AUC p=0.031, Ca2+ p=0.034, females contralateral HbO2 AUC p=0.12, Ca2+ p=0.32; Figure 3-1, Table 1). In the ipsilateral ROI response, FLX and VEH groups analyzed as distinct male or female populations were not significantly different but females displayed a trend toward increased ipsilateral ROI AUC in the HbO2 signal and demonstrated a significant increase in ipsilateral AUC response in the calcium signal (Wilcoxon rank sum test; males HbO2 ipsilateral AUC p=0.15, Ca2+ p=0.34, females HbO2 ipsilateral AUC p=0.075, Ca2+ p=0.0042; Figure 3-1, Table 1).”

3. Added the following text to the discussion:

a. “We utilized both sexes of animals so that our results would be more generalizable, and although our study was not deliberately powered for analysis by sex, post hoc the trends seen in the evoked response to forepaw stimulation suggest that female and male brains respond similarly to perinatal SSRI exposure. It is true that in both the hemodynamic and calcium measures, differences in males' contralateral response and females' ipsilateral response appeared more prominent, suggesting that further studies focused on sex differences could be a promising research direction.”

2. Clarification on why this study design included both pre- and post-natal SSRI treatment would be helpful.

We thank the reviewers for pointing out the missing rationale for the pre- and postnatal SSRI treatment. In brief, the pre/post treatment provides a longer treatment window for an increased integrated effect. We have added the following to the Methods section:

“This exposure time period and dosage were chosen in order to replicate the effects of usage of the maximum recommended human dose of FLX (Marken, 2000) and to mirror the period of human pregnancy and early life breastfeeding, as the final trimester of human pregnancy is equivalent to the first week of life in a murine mouse model, and the remaining week (postnatal day 8-14) during which the pups were exposed through their mother's milk mirrors a post-birth breastfeeding period.”

3. “Five cohorts were acquired by using two different settings. Did authors consider the signal difference between cohorts 1-2 and 3-5 because all of mice of five cohorts were directly used in the group analysis?”

We thank the reviewers for bringing up the different imaging system models used for cohorts 1-2 and 3-5 and whether the different machines might cause a signal difference. We accounted for this possible discrepancy in the original submission's forepaw stimulation analysis by using cohorts 1-2 to define our regions of interest and then exclusively using cohorts 3-5 to perform the analysis of regional intensity and activation across time in Figures 2-4. When using all 5 cohorts for Figure 1's HbO2 maps, the correlation coefficient calculation also mathematically accounts for and eliminates any signal difference issues before group averaging is performed. To clarify the use of cohorts in stimulation analysis, we have added the following text to the Methods:

“Resting-state analyses include hemodynamic data from cohorts 1-5 to maximize sample size, and calcium data from the GCaMP-positive cohorts, Cohorts 3-5. The forepaw stimulation analyses used Cohorts 1 and 2 to define regions of interests, which were then used to subsequently analyze the hemodynamic and calcium response data from Cohorts 3-5.”

4. Figure 1B shows that forepaw stimulation of 15 minutes was conducted before the resting state recording of 30 minutes. In general, in order to avoid effects of the task stimulation, the rest state scan precedes all of task scans. It is not clear why the resting state measurement was not acquired before the electrical stimulation in the present study. Could authors supply a brief explanation?

The order of task vs rest imaging can vary depending on the goals of human connectome fMRI studies. Residual effects of task stimulation, if present, have been previously shown in human fMRI literature to not prevent the extraction of meaningful resting-state data between or after task periods (Jurkiewicz MT, Crawley AP, Mikulis DJ (2018) Is Rest Really Rest? Resting-State Functional Connectivity During Rest and Motor Task Paradigms. Brain Connectivity 8:268-275). The following text was added in the Methods section:

“The block design was modeled on existing fMRI and functional optical imaging stimulation studies, in which mixed blocks of stimulation and resting state are common. Although some fMRI studies have shown a subtle alteration of resting state due to prior motor learning tasks (Albert et al., 2009; Zhang et al., 2014), these effects have not been explored as extensively with mice and OIS. In either case, in this study we used the same design structure for both groups of mice and thus the effect or lack of effect would equally influence both groups.”

5. The intervals of the ketamine injection to forepaw stimulation and forepaw stimulation to resting state were not mentioned in either the Figure 1 legend or the methods section. Are these intervals of each mouse same? Particularly, is the interval of the forepaw stimulation to resting state long enough to eliminate the effects of stimulations for the resting state functional connectivity?

The interval between each mouse's response to ketamine injection and the start of forepaw stimulation was uniform across mice, as well as the interval between forepaw stimulation end and the beginning of resting state data collection. We neglected to clearly state that we always control for possible data artifacts lingering from stimulations by performing a data quality check on each 5-minute resting state period within the 30-minute collection interval. We did not observe a higher incidence of poor quality data falling below our elimination threshold for the first run post-stimulation than any of the other 5-minute resting state periods.

The following information was added to the Methods section:

1. “Insertion of the mouse into the imaging apparatus was not initiated until full transition to the anesthetized state was observed, as measured by the absence of whisker movement or a response to a toe-pinch stimulus.”

2. “The first resting state run for each mouse was initiated approximately three minutes following any stimulus application from previous data collection blocks.”

6. Was the binary brain mask created for each mouse before the co-registration processing? Could authors add an example figure to show the boundary of the binary brain mask and corresponding landmarks?

We have clarified that the binary brain mask was created for each mouse, and how the mask is used within the processing pipeline in the Methods section:

“Following a previously published approach (White et al., 2011), the co-registration processing began with defining an individual-specific binary brain mask that was created for each mouse and used to eliminate pixels not containing brain tissue (Figure 1-1). Within the mouse images we manually labelled the locations of lambda, where superior colliculus and cerebrum intersect at midline, and the anterior suture between the olfactory bulb and cerebrum at midline. These coordinates were then used to co-register all mice to common Paxinos atlas space (Franklin and Paxinos, 2008) using a two-dimensional affine transformation.”

7. Authors need to add more details about the homotopic contralateral functional connectivity approach. It is not clear why Figure C-J only shows the averaged functional connectivity map of the left hemisphere. Was the contralateral functional connectivity of each pixel calculated in this analysis? Could authors also show the averaged map of the right hemisphere as shown in Figures 2 and 2-1?

We appreciate the need for clarifying this figure. In Figure 2 we are computing the contralateral homotopic functional connectivity of each pixel pair between left and right hemispheres. Because the functional connections are between pairs of locations that are homotopic, in the same locations, about midline, the mapping is symmetric for the left and right hemispheres. Mathematically, the right-side map is an exact mirror image of the left side map. We have added a clarifying statement to the Methods to explain why Figure 1C-J show averaged maps of the left hemisphere only.

“Because the homotopic contralateral connectivity methods compare pairs of pixels that are reflections of each other between the left and right hemisphere, the left and right hemisphere maps are mirror images about midline. We therefore report via heat map the connectivity coefficients in the left hemisphere and display the dorsal cortical surface and its landmarks on the right hemisphere, to provide anatomical context when viewing the values displayed across midline.”

8. Authors need to add more detail of the Bonferroni correction approach. What are the uncorrected p values and the amount of the one-tailed Wilcoxon rank sum or student test in the calculation of Bonferroni correction?

We added the following in the Methods section:

“For HCFC analyses, a T-test was performed for each brain pixel pair and represented by a heat map value in that pixel within the cortical image. With the data smoothed by a Gaussian blur with full width at half maximum of 0.096 mm, and a field of view of 22.9 mm2, an estimated 792 independent measures exist. The minimum uncorrected p-value=0.0015 (HbO2) or 6.85E-4 (Ca2+), but with a Bonferroni correction, performed for 792 tests, the corrected p-value threshold=6E-5, and therefore none of the comparisons survive correction as they are at least an order of magnitude too high.”

9. Data from five independent cohorts (Cohort 1: n=7 FLX, 8 VEH; Cohort 2: n=8,10; Cohort 3: n=9,8; Cohort 4: n=9,8; Cohort 5: n=17,11, total 50 FLX and 45 VEH) were acquired in the present study. However, data from a fewer number of mice of are reported. Authors should add a detailed rationale for why some mice were discarded for each analysis. For example, in Figure 1, 48 FLX and 42 VEH mice were used in C-F, and 34 FLX and 25 VEH mice were used in G-J.

We have added the following clarifying statement in the Methods section (Image Data Processing and Analysis):

“Figure 1C-F includes more mice than G-J because C-F are hemodynamic measures and include the data of cohorts 1-5 to include the maximum sample size. Figure 1G-J included less mice because it is a calcium measure and cohorts 1-2 are GCaMP-negative and therefore have no fluorophore excitation to record and average into the functional connectivity maps.”

10. 15 minutes and 30 minutes are acquired for the forepaw stimulation and the resting state recording, respectively, for each mouse. Could authors also supply a brief summary about how many minutes' data were used after the quality control and pre-processing procedure?

We have added a statement indicating that 5 minutes minimum were used of resting state, and 5 minutes minimum were averaged together to produce stimulation data.

“After quality control, for resting state analysis an average of 70% of the data per mouse was used, with a minimum requirement of five minutes of data from each mouse. For stimulation an average of 80% of the data per mouse was used, with a minimum of 5 minutes (5 stimulation presentations) of data from each mouse.”

11. The authors state on page 14, that “as expected, the observed region of greatest activation was the forepaw region of the somatosensory cortex”. Please show data for the levels of activation. How does this compare with other SS1 regions, such as the barrel field? Is there minimal activation in other SS1 regions over forepaw?

We thank the reviewers for pointing out the necessity of a figure describing the anatomical localization of the signal. We have added Extended Figure 2-2 with a Paxinos atlas-based map of cortical regions and clarified the text in the Results section:

“As expected, the observed region of greatest activation was the forepaw region of the somatosensory cortex, as defined by the cortical regions in the Paxinos histological atlas (Franklin and Paxinos, 2008) and adapted to be a dorsal-view cortical map in White, et al. (2011) (Figure 2-2).”

Discussion and interpretation

12. It is unclear what “a decrease in contralateral activation and comparative increase in ipsilateral activity means”, as there were no changes in baseline. What are the speculated consequences of this result? Could this be due to handedness of the mice being compared? Were the mice tested for this before the imaging or forepaw stimulation?

We thank the reviewers for pointing out this confusing wording regarding the abnormal stimulation response in the cortical regions and its consequences. We have clarified this point about activation regions in the Results:

“This decrease in contralateral activation and comparative increase in ipsilateral cortical activity during and immediately following stimulation was observed in the 3 GCaMP6f cohorts' calcium dynamics as well (Figure 2E-H), confirming that underlying neural activity is altered upon somatosensory stimulation.”

We have also clarified this by modifying the text in the Discussion to read as follows:

“Suppression of activity in ipsilateral somatosensory cortex during stimulus presentation plays a role in the successful execution of sensory gating (Staines et al., 2002) and, by extension, the ability to process and react to sensory stimuli in an organism's environment as needed. Abnormal ipsilateral cortical inhibition suggests that developmental SSRI exposure causes long-term sensory gating and processing abnormalities.”

We did not test mice for handedness.

13. Left forepaw was stimulated in these assays. Would the authors expect to observe similar, reverse results if the right paw was stimulated? This could point at the level of activation vs. morphological/connectivity issue discussed in the paper.

To elaborate on why we would expect the same effect if right forepaw were stimulated, we have added the following explanation of the stimulated paw's importance in the Results:

The left forepaw was exclusively stimulated in these assays, but similar effects would be expected if right forepaw was stimulated instead, due to the equal number of left- and right-handed individuals in the C57Bl/6J population (Collins, 1968).

14. Reviewers recommend analyzing contralateral and ipsilateral imaging data per individual mouse to determine if these results might have consequences to individual mice in processing tactile information - which is one of the points the authors suggest for implications in variability in humans.

We agree that comparison of individual's imaging metrics with tactile behavioral data would have important implications regarding the variability of individual response to SSRI exposure. We have added the following to the Discussion:

“Comparison of individuals' somatosensory stimulation response in the cortex with their performance on tactile behavioral assays such as the von Frey test of somatosensation should also be considered, as it has previously been shown to be sensitive to developmental SSRI exposure perturbations (Maloney et al., 2018), and has the potential to provide insights into individual variability in populations developmentally exposed to SSRIs.”

15. fMRI is very powerful non-invasive imaging approach, which can complement the invasive 3D BOLD data for resting state functional connectivity and forepaw stimulation approach. Could authors add some discussion about the fMRI and optical imaging approach? Will authors plan to run further fMRI studies in the future?

We agree that these studies should be replicated with fMRI in mice to improve insights into the functional neurophenotype of this mouse model. We have added the following in the Discussion:

“In future studies it will be important to consider using functional MRI in mice, to evaluate whether these OIS hemodynamic findings are replicated using fMRI's blood oxygen level-dependent (BOLD) signal and potentially explore subcortical elements of the circuit in which the abnormal evoked somatosensory response was observed.”

Minor

16. Pg.24: “gaiting” was mentioned a few times. Is “gating” the intended word?

Yes, we thank the reviewers for pointing out this error and have corrected it.

We appreciate the comments provided by the editor and reviewers and believe these revisions strengthen the manuscript.
