# Supplementary figures and images for: Maternal Fluoxetine Exposure Alters Cortical Hemodynamic and Calcium Response of Offspring to Somatosensory Stimuli
Source: eNeuro. 2019 Dec 19;6(6):ENEURO.0238-19.2019. doi: 10.1523/ENEURO.0238-19.2019 (PMC6978917; doi:10.1523/ENEURO.0238-19.2019)

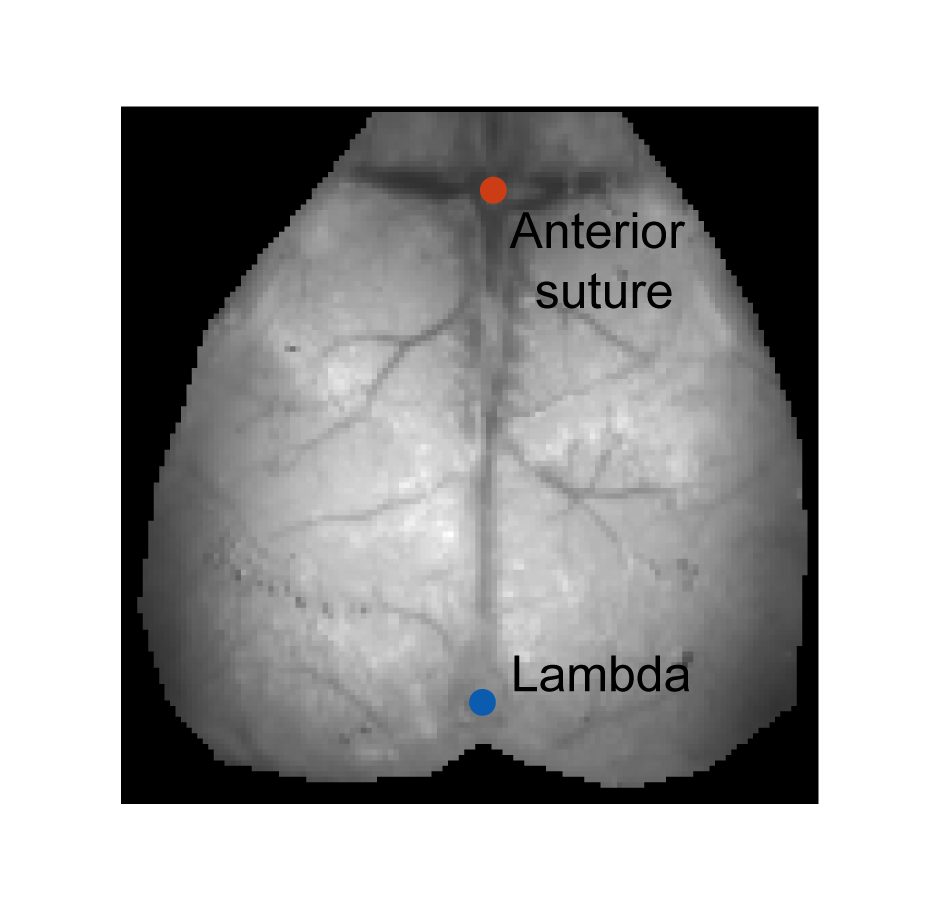

Supplement: Extended Data Figure 1-1 — Image analysis is performed after excluding pixels not containing brain tissue and identifying landmarks to perform two-dimensional affine transformation. Representative mouse brain displaying mask excluding non-brain pixels within the 128 × 128-pixel field of view. Red denotes where the anterior suture between olfactory bulb and cerebrum intersects the midline. Blue denotes lambda, where superior colliculus and cerebrum intersect at midline. Download Figure 1-1, TIF file. [file sup_enu-eN-NWR-0238-19-s02.tif]

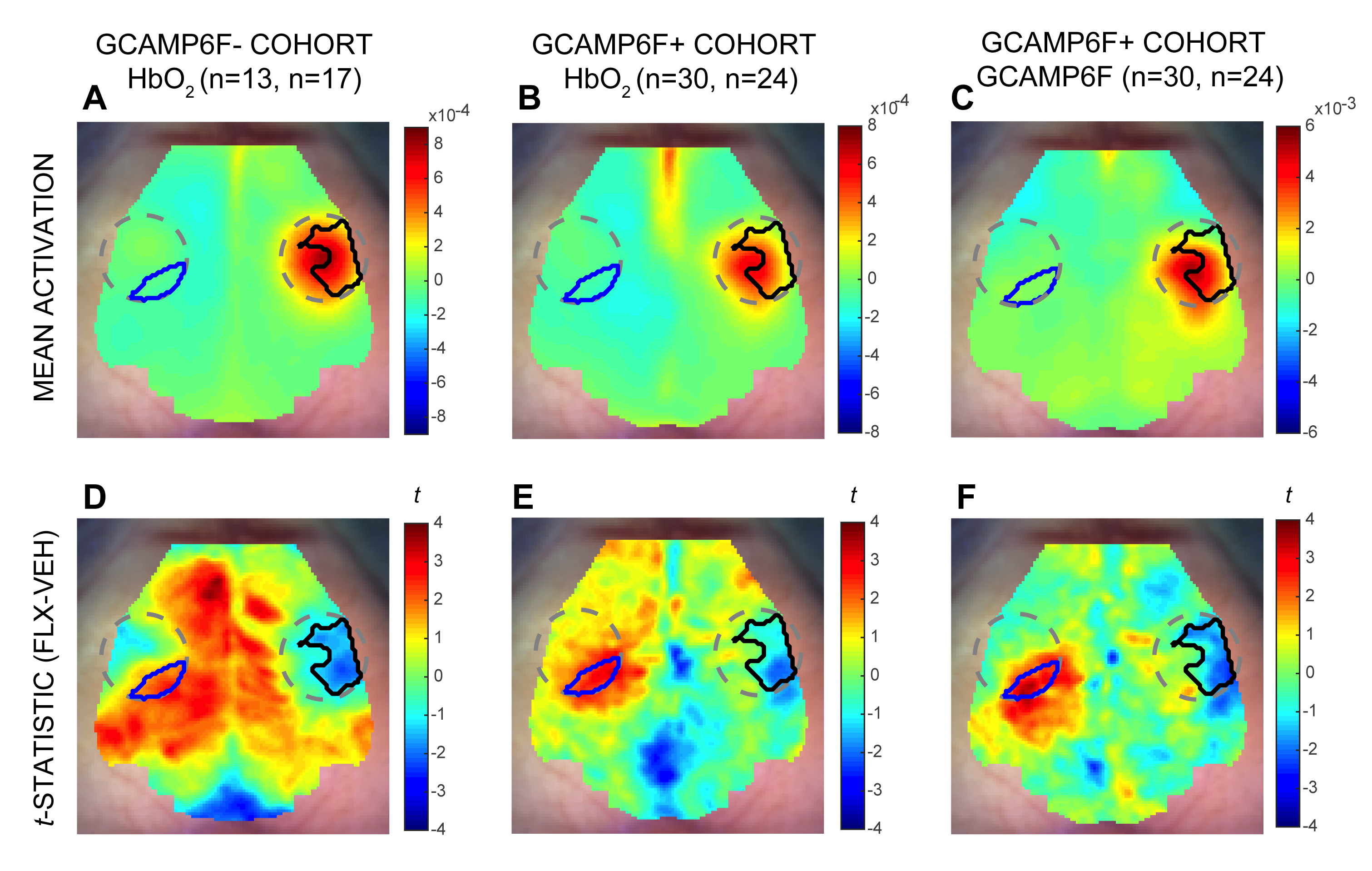

Supplement: Extended Data Figure 2-1 — Contralateral and ipsilateral ROIs defined by half-maximum or half-minimum pixels from the t statistic map of GCaMP-negative cohorts 1 and 2. A, Mean HbO2 of the GCaMP6f-negative (cohorts 1 and 2) mice at end of forepaw stimulation (t = 10 s; FLX n = 13, VEH n = 17). Grey circles delineate the regions of radius 15 pixels from which half-minimum t statistic pixels were selected for the contralateral ROI (black region) and half-maximum t statistic pixels were selected for the ipsilateral ROI (blue region). B, Mean HbO2 of the GCaMP6f-positive cohorts (cohorts 3–5) at end of forepaw stimulation (t = 10 s) with predefined ROIs overlaid (FLX n = 30, VEH n = 24). C, Mean GCaMP6f calcium (ΔF) of the GCaMP6f-positive cohorts (cohorts 3–5) at end of forepaw stimulation (t = 10 s) with predefined ROIs overlaid (FLX n = 30, VEH n = 24). D, HbO2 t statistic map of the GCaMP6f-negative (cohorts 1 and 2) mice at end of forepaw stimulation (t = 10 s; FLX n = 13, VEH n = 17). Grey circles delineate the regions of radius 15 pixels from which half-minimum t statistic pixels were selected for the contralateral ROI (black region) and half-maximum t statistic pixels were selected for the ipsilateral ROI (blue region). E, HbO2 t statistic map of the GCaMP6f-positive cohorts (cohorts 3–5) at end of forepaw stimulation (t = 10 s; FLX n = 30, VEH n = 24). F, GCaMP6f calcium t statistic map of the GCaMP6f-positive cohorts (cohorts 3–5) at end of forepaw stimulation (t = 10 s; FLX n = 30, VEH n = 24). Download Figure 2-1, TIF file. [file sup_enu-eN-NWR-0238-19-s03.tif]

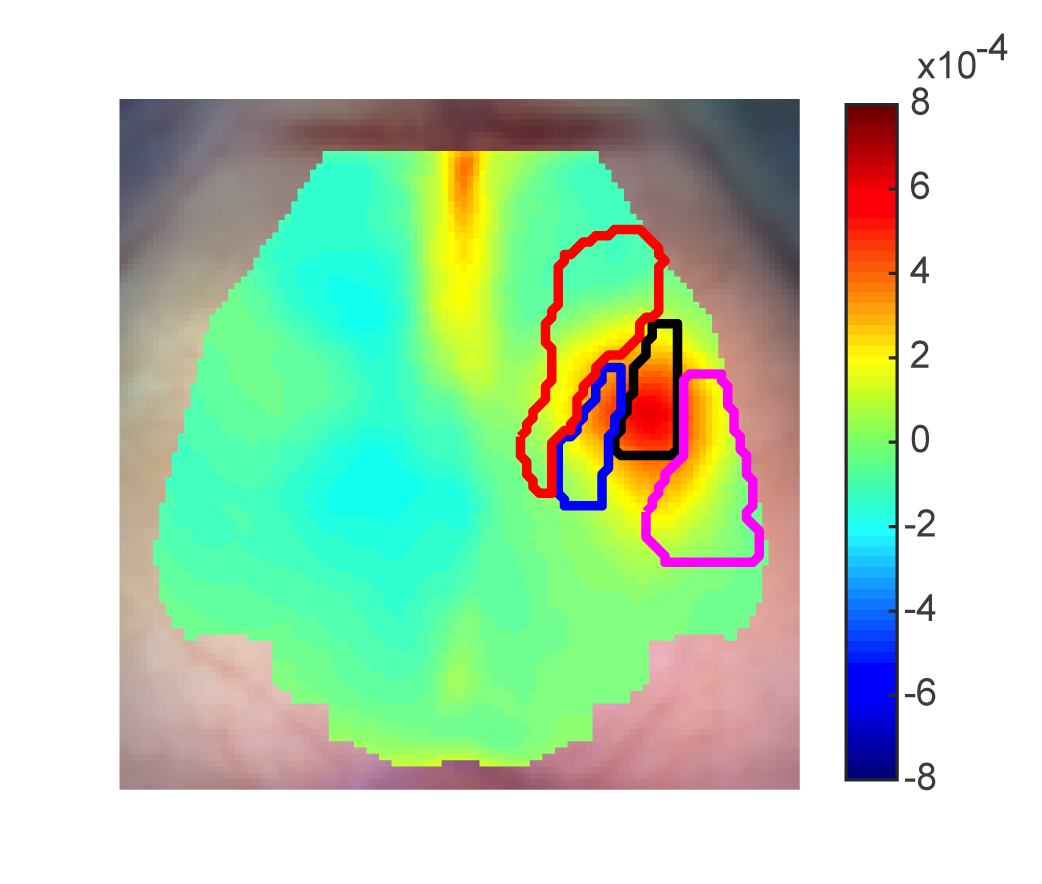

Supplement: Extended Data Figure 2-2 — Left forepaw stimulation results in activation of the right cortical forepaw region, as defined by the Paxinos atlas. Mean HbO2 stimulation averaged across all mice and groups with forepaw (black), M1 (red), hindpaw (blue), and whisker barrel (magenta) functional region boundaries mapped on the mouse cortex. Download Figure 2-2, TIF file. [file sup_enu-eN-NWR-0238-19-s04.tif]

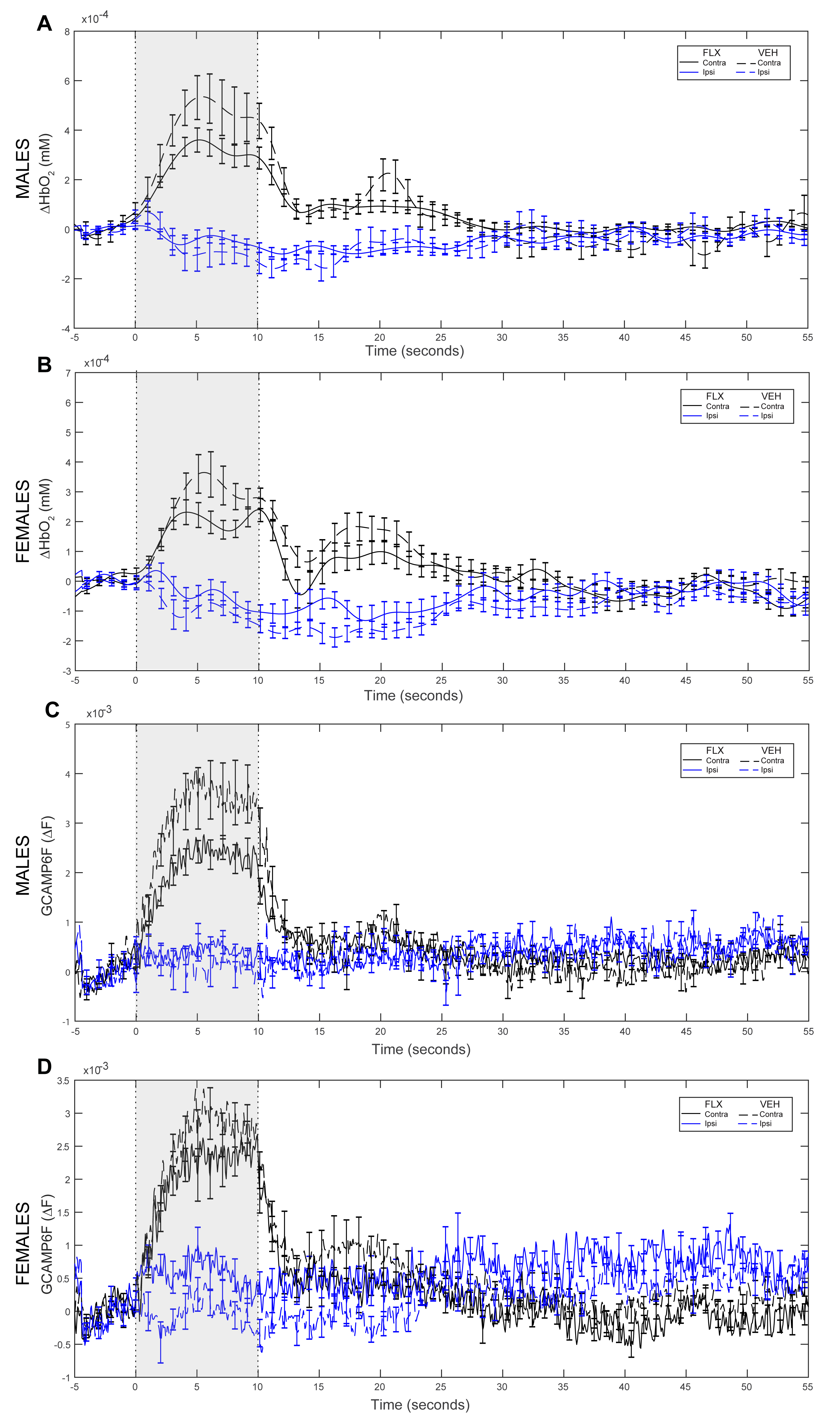

Supplement: Extended Data Figure 3-1 — HbO2 and Ca2+ response amplitude is altered in both males and female FLX mice during forepaw stimulation. A, Block-averaged time trace of mean ΔHbO2 response in males to forepaw stimulation in each ROI (FLX n = 18, VEH n = 11; black = contralateral, blue = ipsilateral). Left forepaw stimulation at 3 Hz applied t = 0 s to t = 10 s. B, Block-averaged time trace of mean ΔHbO2 response in females to forepaw stimulation in each ROI (FLX n = 12, VEH n = 13; black = contralateral, blue = ipsilateral). Left forepaw stimulation at 3 Hz applied t = 0 s to t = 10 s. C, Block-averaged time trace of GCaMP6 Ca2+ response in males to forepaw stimulation in each ROI (FLX n = 18, VEH n = 11; black = contralateral, blue = ipsilateral). Left forepaw stimulation at 3 Hz applied t = 0 s to t = 10 s. D, Block-averaged time trace of GCaMP6 Ca2+ response in females to forepaw stimulation in each ROI (FLX n = 12, VEH n = 13; black = contralateral, blue = ipsilateral). Left forepaw stimulation at 3 Hz applied t = 0 s to t = 10 s. Download Figure 3-1, TIF file. [file sup_enu-eN-NWR-0238-19-s05.tif]
